# YY1 regulation by miR-124-3p promotes Th17 cell pathogenicity through interaction with T-bet in rheumatoid arthritis

**DOI:** 10.1172/jci.insight.149985

**Published:** 2021-11-22

**Authors:** Jinpiao Lin, Jifeng Tang, Junyu Lin, Yujue He, Ziqing Yu, Renquan Jiang, Bin Yang, Qishui Ou

**Affiliations:** 1 Department of Laboratory Medicine, Gene Diagnosis Research Center, and; 2 Fujian Key Laboratory of Laboratory Medicine, The First Affiliated Hospital, Fujian Medical University, Fuzhou, China.; 3 The First Affiliated Hospital of Fujian Medical University, Fuzhou, China.

**Keywords:** Autoimmunity, Immunology, Arthritis, Autoimmune diseases, T cells

## Abstract

Th17 cells are involved in rheumatoid arthritis (RA) pathogenesis. Our previous studies have revealed that transcription factor Yin Yang 1 (YY1) plays an important role in the pathogenic mechanisms of RA. However, whether YY1 has any role in Th17 cell pathogenicity and what molecular regulatory mechanism is involved remain poorly understood. Here, we found the proportion of pathogenic Th17 (pTh17) cells was significantly higher in RA than in control individuals and showed a potential relationship with YY1 expression. In addition, we also observed YY1 expression was increased in pTh17, and the pTh17 differentiation was hampered by YY1 knockdown. Consistently, knockdown of YY1 decreased the proportion of pTh17 cells and attenuated joint inflammation in collagen-induced arthritis mice. Mechanistically, YY1 could regulate the pathogenicity of Th17 cells through binding to the promoter region of transcription factor T-bet and interacting with T-bet protein. This function of YY1 for promoting pTh17 differentiation was specific to Th17 cells and not to Th1 cells. Moreover, we found miR-124-3p negatively correlated with YY1 in RA patients, and it could bind to 3′-UTR regions of YY1 to inhibit the posttranscriptional translation of YY1. Altogether, these findings indicate YY1 regulation by miR-124-3p could specifically promote Th17 cell pathogenicity in part through interaction with T-bet, and these findings present promising therapeutic targets in RA.

## Graphical abstract

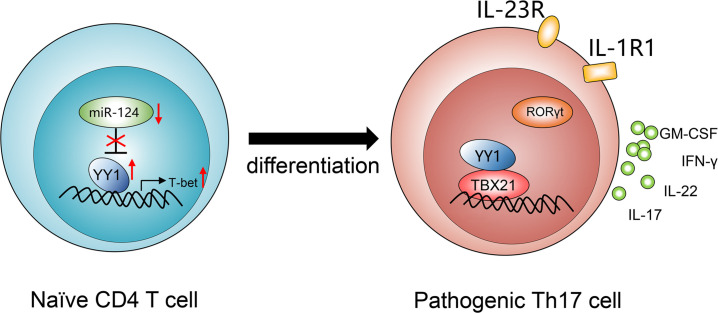

## Introduction

Rheumatoid arthritis (RA) is a chronic autoimmune disease characterized by swollen joints and synovial inflammation as well as cartilage and bone erosion (1). Although the underlying pathophysiological mechanisms of RA are still largely unknown as it involves a complex interaction between genes and environment (2), increasing data demonstrate that the infiltration of inflammatory cells is one of the main etiological factors of RA. The abnormal activity of immune cells, such as Th cells — including Th17 cells — B cells, and macrophages, is responsible for the pathogenesis of RA (3–5).

Th17 cells are a subset of CD4^+^ T cells and associated with the pathogenesis of various autoimmune disorders. Emerging evidence indicates that Th17 cells are heterogeneous and able to differentiate into diverse subsets with different phenotypes according to their pathogenicity. Th17 cells induced by TGF-β1/IL-6 (known as nonpathogenic Th17, non-pTh17) benefit the homeostasis of tissues in certain conditions while those induced by IL-1β/IL-6/IL-23 (known as pathogenic Th17, pTh17) promote the tissue inflammation and pathogenesis of RA (5–8). Pathogenic Th17 can be distinguished from nonpathogenic Th17 based on the different molecular programs. For example, pTh17 expresses high levels of the Th1-associated molecules, such as T-box transcription factor 21 (T-bet), IFN-γ, and CXCR3. The increased expression of proinflammatory genes, such as *Stat4*, *Runx1*, *Csf2*, *Il23r*, *Il17a*, and *Il17f*, and decreased expression of immune-suppressive genes, such as *Ahr*, *Il4*, *Cd5l*, and *Il10*, are also remarkable features of pTh17 cell phenotype. The distinct molecular program of pTh17 makes it display various pathogenic properties (9, 10). However, the precise mechanism of the generation and regulation of pTh17 is still largely unknown.

Yin Yang 1 (YY1) is an evolutionarily conserved transcription factor and member of the GLI-Kruppel family belonging to zinc finger DNA binding proteins. As implied by its name, YY1 activates or represses different eukaryotic genes depending on promoter environment, chromatin structure, and interacting factors (11, 12). Accumulating data have shown that YY1 is implicated in various homeostatic processes and pathological processes of disease, especially in cancer (13). Moreover, YY1 regulates diverse immunological activities and plays critical roles in B cell development, maturation, and immunoglobulin (Ig) class-switch recombination (14, 15). Our previous work has illustrated that YY1 is involved in the RA inflammation process and Th17 differentiation by regulation of IL-6 (16) and IL-8 (17). However, we did not further explore which Th17 cell subsets YY1 exactly affected, and whether YY1 is associated with regulation of pTh17 cells in RA remains unknown.

MicroRNAs (miRNAs), endogenous, small (~22 nucleotides), noncoding RNAs that regulate gene expression at the posttranscriptional level, have been demonstrated to participate in a wide range of physiological and pathophysiological processes (18). By binding to the 3′-untranslated region (3′-UTR) of the target mRNAs, miRNAs mediate posttranscriptional repression through transcript degradation or translational inhibition (19). Moreover, accumulating evidence shows that miRNAs are responsible for the pathogenesis of RA (20), and several miRNAs have been identified as playing roles in induction and regulation of pTh17 cells. For example, the miRNA-183-96-182 cluster contributes to pathogenicity of Th17 cells by inhibiting FOXO1 expression (21) while miR-155 promotes pTh17 cell differentiation by suppressing the inhibitory effects of Jumonji- and ARID-domain-containing protein 2 (22). Based on these data, we reason that there must be certain miRNAs the could bind YY1 3′-UTR to regulate YY1 function, and this process may be involved in the pathogenicity of Th17 cells.

In the present study, an increased proportion of pTh17 cells was observed in patients with RA. Knocking down YY1 expression decreased the proportion of pTh17 cells in vivo and in vitro. Moreover, the underlying molecular mechanism of YY1 in pTh17 differentiation was further investigated. We found that YY1 could bind to the promoter region of the T-bet gene to facilitate the transcription process and directly interact with T-bet protein to control downstream reactions. However, these findings had not been replicated in Th1 differentiation. Furthermore, we confirmed in subsequent in silico research that miR-124-3p is predicted to target the YY1 3′-UTR to repress its expression. In patients with RA, miR-124-3p was markedly downregulated and negatively correlated with YY1 expression levels in purified CD4^+^ cells sorted from peripheral blood mononuclear cells (PBMCs). Altogether, our study reveals that YY1 regulation by miR-124-3p specifically contributed to the differentiation of pTh17 cells partially via promoting T-bet gene transcription and interacting with T-bet protein. Treatment strategies targeting YY1 or miR-124-3p may represent a new option for management of RA.

## Results

### Pathogenic Th17 cells increase in patients with RA and show potential relationship with YY1 expression.

**Figure 1:**
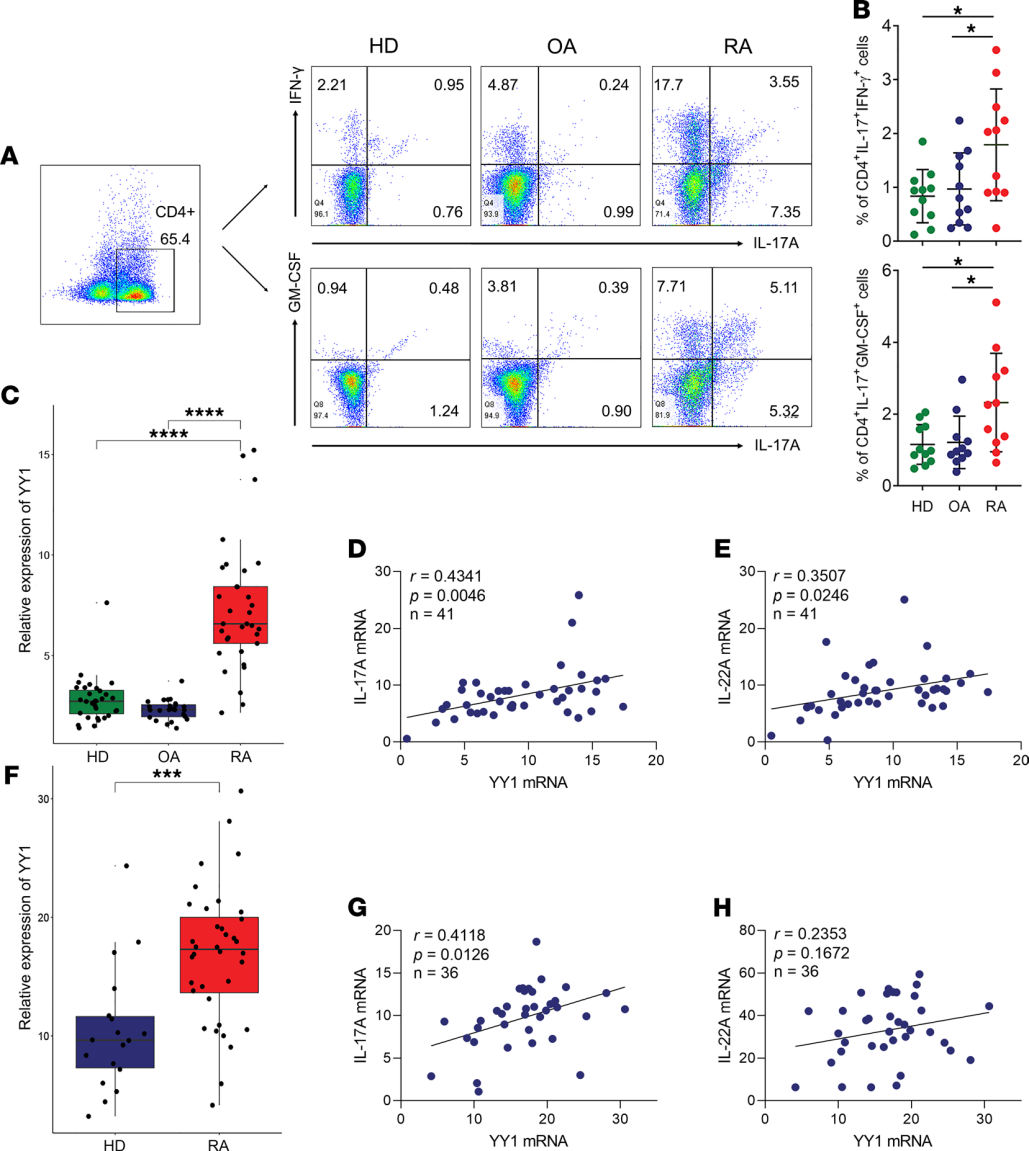
Increased pTh17 cells have potential relationship with YY1 expression in RA. (**A** and **B**) Representative images of flow cytometry results for the proportion of CD4^+^IL-17A^+^IFN-γ^+^ cells and CD4^+^IL-17A^+^GM-CSF^+^ cells in PBMCs of patients with RA and OA as well as HDs. Data presented as mean ± SD (*n* = 11). *P < 0.05 (ANOVA). (**C**) The relative gene expression levels of YY1 in PBMCs of patients with RA (*n* = 33) and OA (*n* = 24) as well as HDs (*n* = 32). Data presented as box-and-whisker plot (The line within the box and the bounds of the box represent median and interquartile range, respectively. The whiskers denote the 25th percentile minus 1.5 interquartile range and 75th percentile plus 1.5 interquartile range. The points, which are outside the whisker, represent outliers). ***P < 0.001, ****P < 0.0001 (ANOVA). (**D** and **E**) Analysis of the correlation of IL-17A or IL-22 mRNA and YY1 mRNA in PBMCs of RA patients (*n* = 41, Pearson correlation). (**F**) The relative gene expression levels of YY1 in purified CD4^+^ T cells of patients with RA (*n* = 36) and HD (*n* = 18). Data presented as box-and-whisker plot (The meaning of the symbols is the same as **C**). ***P < 0.001 (Student’s *t* test). (**G** and **H**) Analysis of the correlation of IL-17A or IL-22 mRNA and YY1 mRNA in purified CD4^+^ T cells of RA patients (*n* = 36, Pearson correlation).

To explore the roles of YY1 and pTh17 cells in RA, we first examined the proportion of pTh17 cells in PBMCs of patients with RA using flow cytometry analyses. A representative scheme showing the flow cytometry gating strategy used for the analysis of pTh17 cells defined by CD4^+^IL-17A^+^IFN-γ^+^ or CD4^+^IL-17A^+^GM-CSF^+^ cells (21, 23) is provided in Supplemental Figure 1; supplemental material available online with this article; https://doi.org/10.1172/jci.insight.149985DS1 As expected, the results showed that the proportions of CD4^+^IL-17A^+^IFN-γ^+^ cells and CD4^+^IL-17A^+^GM-CSF^+^ cells were elevated in PBMCs of patients with RA compared with patients with osteoarthritis (OA) and healthy donors (HDs) (Figure 1, A and B). Similarly, the expression levels of YY1 mRNA in PBMCs of 3 cohorts were also detected. As shown in Figure 1C, increasing YY1 mRNA levels were confirmed in PBMCs of patients with RA. Further analysis to correlate YY1 mRNA expression with Th17-related cytokines’ mRNA in PBMCs of patients with RA demonstrated a positive correlation between YY1 and IL-17A (Figure 1D) or IL-22 (Figure 1E). Moreover, CD4^+^ cells were purified by negative selection with magnetic beads from PBMCs of patients with RA for further analysis. Similar to previous observations, we found that YY1 mRNA expression was upregulated in purified CD4^+^ cells of patients with RA compared with HD (Figure 1F), and correlation analysis revealed there was still a positive correlation between YY1 and IL-17A (Figure 1G) yet no significant correlation between YY1 and IL-22 (Figure 1H) in purified CD4^+^ cells from patients with RA. These results implied that increased pTh17 cells may be related to the high expression of YY1 in patients with RA.

### YY1 was involved in pTh17 cell differentiation.

**Figure 2:**
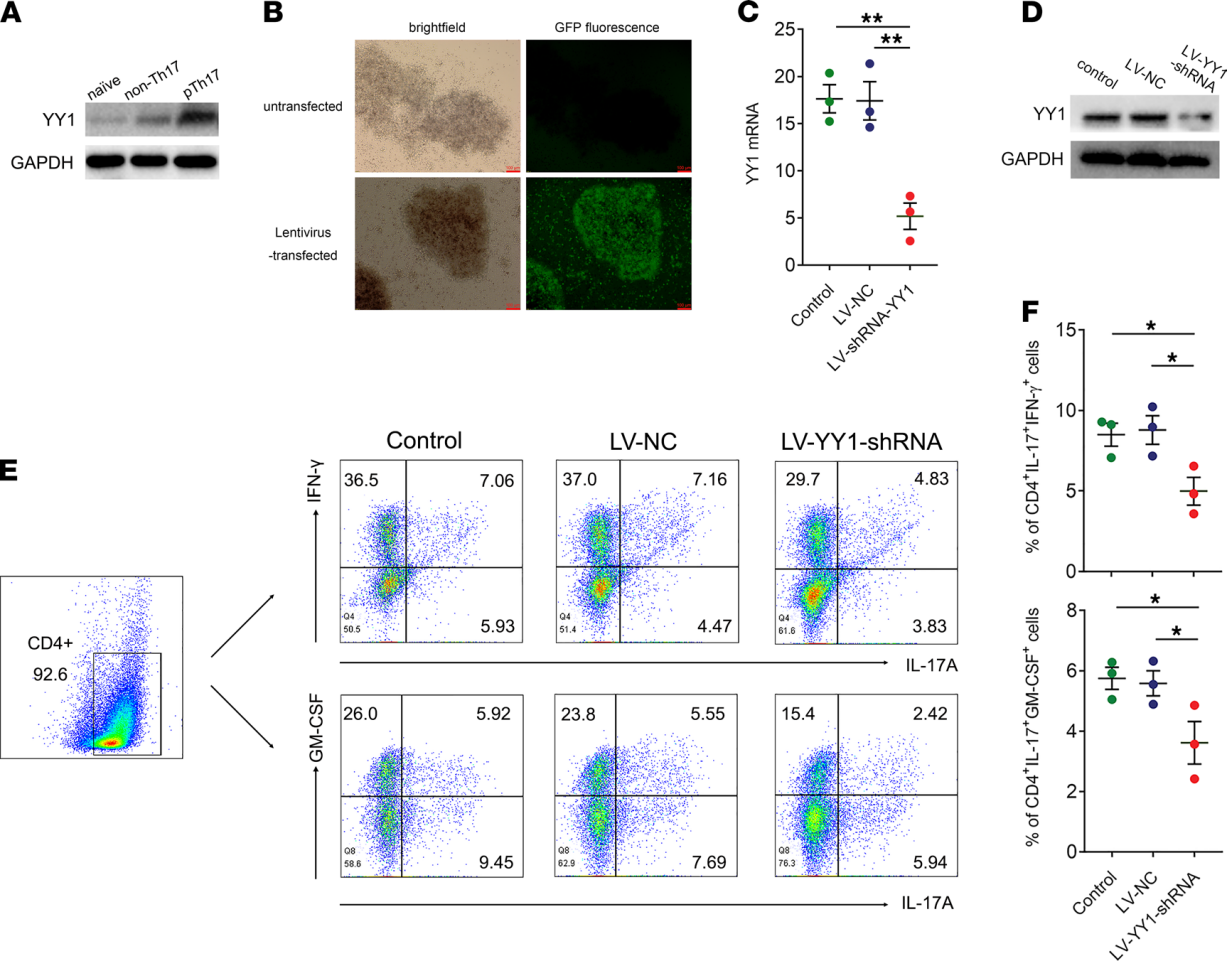
YY1 is involved in pTh17 cell differentiation. (**A**) Representative Western blots of YY1 in an ex vivo Th17 subset differentiation system including unpolarized, non–pTh17-polarized, and pTh17-polarized groups (at least 3 independent experiments). (**B**) Representative fluorescence images for transfection efficiency of the sh-YY1 lentivirus (scale bars = 100 μm, at least 3 independent experiments). (**C**) The relative expression levels of YY1 for knockdown effect of the sh-YY1 lentivirus (*n* = 3 independent experiments). (**D**) Representative Western blots of YY1 for knockdown effect of the sh-YY1 lentivirus (at least 3 independent experiments). (**E** and **F**) Representative images of flow cytometry results for the proportion of CD4^+^IL-17A^+^IFN-γ^+^ cells and CD4^+^IL-17A^+^GM-CSF^+^ cells in the pTh17-polarized group treated with the sh-YY1 lentivirus. All data presented as mean ± SEM (*n* = 3 of independent experiments). **P < 0.01, *P < 0.05 (ANOVA).

To further confirm the relationships between YY1 and pTh17, an ex vivo experimental system was established to induce Th17 subset differentiation using different combinations of cytokines. We first investigated the protein levels of YY1 in naive CD4^+^ T cells (unpolarized group), in vitro polarized non-pTh17 cells (non-pTh17-polarized group), and in vitro polarized pTh17 cells (pTh17-polarized group). Western blot assay demonstrated that YY1 protein level was increased in the pTh17-polarized group compared with the 2 other groups (Figure 2A), which suggested that YY1 was more likely to play a role in pTh17 instead of non-pTh17. In order to further clarify this, YY1 shRNA lentivirus (LV-YY1-shRNA) was transfected into the pTh17-polarized group to knock down YY1 expression. The lentiviral transfection efficiency and YY1 knockdown effect were examined by fluorescence microscopy (Figure 2B), PCR (Figure 2C), and Western blot (Figure 2D). As shown in Figure 2, E and F, the proportions of CD4^+^IL-17A^+^IFN-γ^+^ cells and CD4^+^IL-17A^+^GM-CSF^+^ cells were decreased in the YY1-knockdown pTh17-polarized group. These results demonstrated that YY1 could promote pTh17 cells’ differentiation in vitro.

### Blocking of YY1 attenuates inflammation and downregulates the pTh17 cell population in collagen-induced arthritis mice.

**Figure 3:**
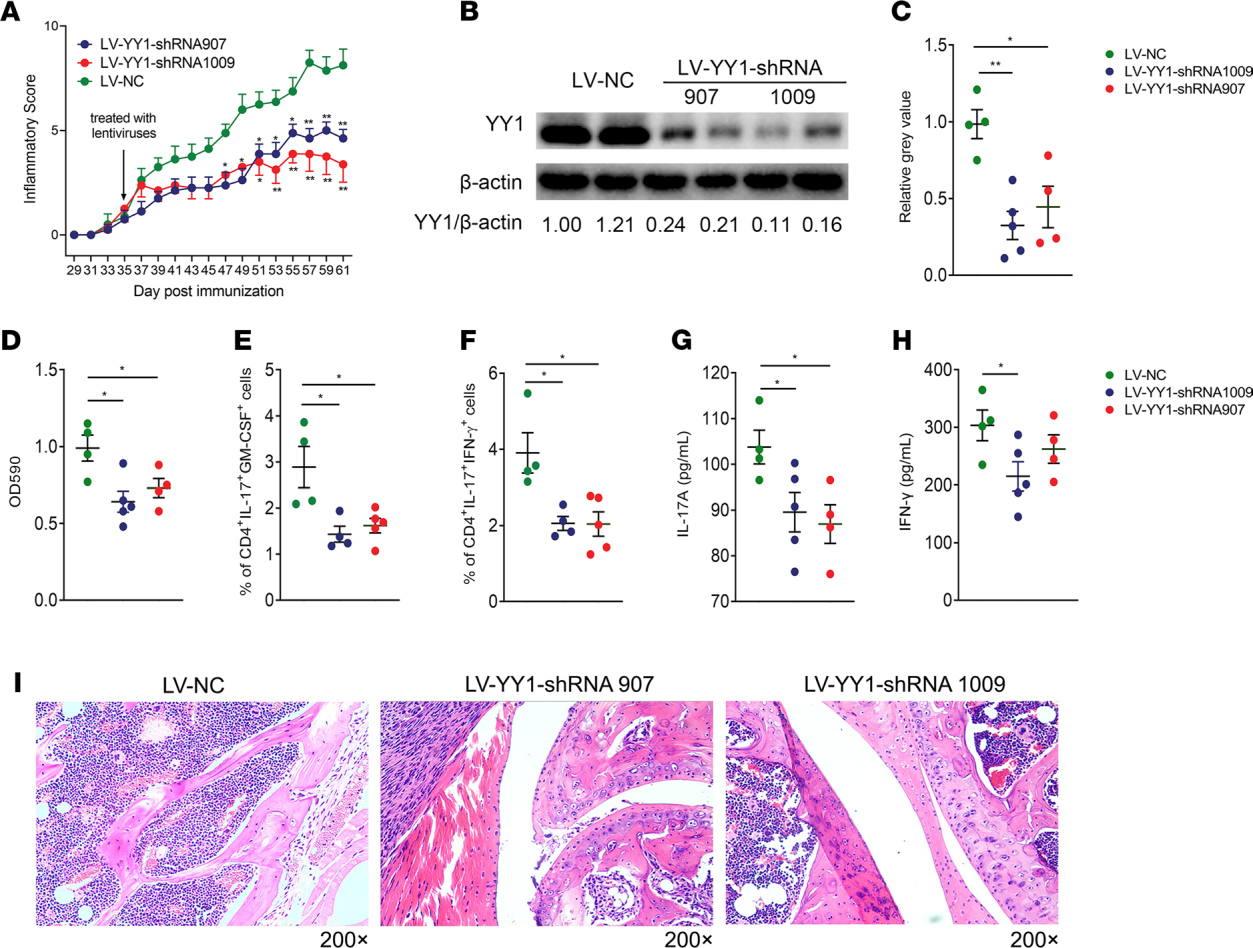
Blocking of YY1 attenuates inflammation and decreases pTh17 cells in CIA mice. (**A**) Inflammatory score of CIA mice treated with LV-YY1-shRNA907 (*n* = 4), LV-YY1-shRNA1009 (*n* = 5), or empty vectors (LV-NC; *n* = 4). (**B**) Representative Western blots of YY1 for knockdown effect of lentivirus-treated CIA mice and (**C**) the grayscale analysis of Western blot analysis results. (**D**) The proliferation assay in vitro of CII-induced mononuclear cells from lentivirus-treated CIA mice. (**E** and **F**) Flow cytometry results for the proportion of CD4^+^IL-17A^+^IFN-γ^+^ cells and CD4^+^IL-17A^+^GM-CSF^+^ cells in PBMCs from lentivirus-treated CIA mice. (**G** and **H**) The levels of serum IL-17A and IFN-γ in lentivirus-treated CIA mice. (**I**) Representative H&E staining images of joint tissue of lentivirus-treated CIA mice. All data presented as mean ± SEM (*n* = 4–5). **P < 0.01, *P < 0.05 (ANOVA).

To validate the results of in vitro experiments, we further constructed a collagen-induced arthritis (CIA) model and treated CIA mice with YY1 shRNA lentivirus (LV-YY1-shRNA-907 or -1009) or control lentivirus (LV-NC) through tail vein injection. In the LV-NC group, no particular side effects were observed in CIA mice compared to the untreated group (data not shown). After the injection of lentiviruses, we noted that the index of joint inflammation was significantly decreased in CIA mice treated with LV-YY1-shRNA (Figure 3A). Moreover, YY1 protein levels were decreased in mononuclear cells derived from CIA mice treated with LV-YY1-shRNA (Figure 3, B and C). Consistently, the proliferation of mononuclear cells isolated from spleens of the CIA mice treated with LV-YY1-shRNA, for collagen type II (CII) antigen challenge in vitro, was significantly inhibited compared with those isolated from the LV-NC–treated group (Figure 3D). PBMCs of CIA mice were also collected to examine the population of pTh17 cells. As shown in Figure 3, E and F, results demonstrated that the population of pTh17 cells decreased in YY1-knockdown CIA mice. In addition, we found that the levels of IL-17A (Figure 3G) and IFN-γ (Figure 3H) in peripheral blood serum of YY1-knockdown CIA mice were downregulated compared with those of controls. Moreover, the cartilage destruction and pannus formation in LV-NC–treated mice were more serious than those in the LV-YY1-shRNA treatment group (Figure 3I). These results suggested that blocking of YY1 could ameliorate the inflammatory reaction and be accompanied by downregulation of pTh17 cells in CIA mice.

### YY1 specifically contributes to pTh17 cell differentiation through affecting T-bet.

**Figure 4:**
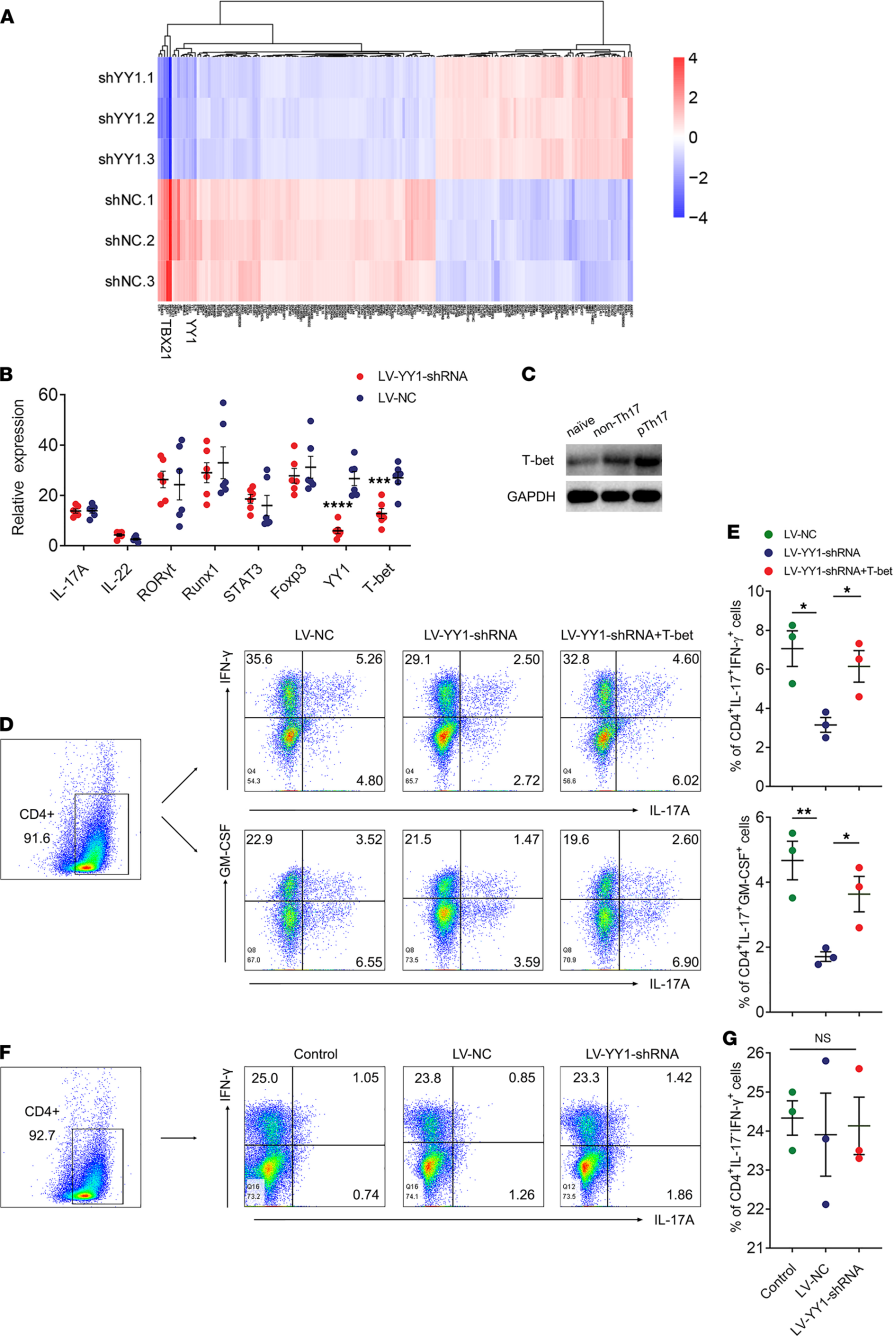
YY1 specifically regulates pTh17 cell differentiation through affecting T-bet. (**A**) Heatmap of clustering analysis of differentially expressed genes of cells treated with the sh-YY1 lentiviruses in pTh17-polarized conditions. (**B**) The relative gene expressions of pTh17-related transcription factors and cytokines in cells from YY1-knockdown pTh17-polarized group. Data presented as mean ± SEM (*n* = 6). ***P < 0.001, ****P < 0.0001 (Student’s *t* test). (**C**) Representative Western blots of T-bet in an ex vivo Th17 subset differentiation system including unpolarized, non–pTh17-polarized, and pTh17-polarized (at least 3 independent experiments). (**D** and **E**) Representative images of flow cytometry results for the proportion of CD4^+^IL-17A^+^IFN-γ^+^ cells and CD4^+^IL-17A^+^GM-CSF^+^ cells in pTh17-polarized group treated with the sh-YY1 lentivirus or cotransfected with sh-YY1 lentivirus and T-bet–overexpressing lentivirus. Data presented as mean ± SEM (*n* = 3 independent experiments). **P < 0.01, *P < 0.05 (ANOVA). (**F** and **G**) Representative images of flow cytometry results for the proportion of CD4^+^IL-17A^+^IFN-γ^+^ cells in Th1-polarized group treated with the sh-YY1 lentivirus. Data presented as mean ± SEM (*n* = 3 independent experiments). *P < 0.05 (ANOVA).

YY1 has been confirmed to be involved in differentiation of Th17 cells by a series of in vitro and in vivo experiments, but it is still unclear how YY1 can affect Th17 cell differentiation in RA. To address this, the mRNA content of cells in the pTh17-polarized group was analyzed by mRNA array to identify the YY1 knockdown effects at the mRNA level. As we know, T cell lineage differentiation is established and maintained via the induction and action of various transcription factors based on the different cytokine milieus; thus, we performed a heatmap analysis on the differentially expressed genes (fold change ≥ 2, corrected *P* ≤ 0.05) annotated as “differentiation” or “transcription” based on Gene Ontology annotations. The mRNA expression profiles showed that the gene expression of TBX21 (encoding T-bet protein), which is the key transcription factor involved in pTh17 differentiation (5, 9), was significantly decreased in the pTh17-polarized group transfected with LV-YY1-shRNA (Figure 4A). To validate the result of the heatmap analysis, quantitative PCR (qPCR) was conducted. Meanwhile, we still included several transcription factors, including RORγt, Runx1, STAT3, and Foxp3, reported to be involved in pTh17 differentiation (5) and certain Th17-related cytokines into the qPCR assay to prevent the missed detection of the mRNA array. The results showed the expression level of T-bet was reduced after YY1 knockdown while no differential expressions were observed in other transcription factors or IL-17A as well as IL-22 (Figure 4B). Moreover, Western blot results suggested T-bet protein levels were similar to the results of YY1 protein levels in the ex vivo Th17 subset differentiation system (Figure 4C).

Subsequently, we constructed T-bet–overexpressing lentivirus and transfected it into the YY1-knockdown pTh17-polarized group. The results revealed that overexpression T-bet rescued the proportions of CD4^+^IL-17A^+^IFN-γ^+^ cells and CD4^+^IL-17A^+^GM-CSF^+^ cells successfully (Figure 4, D and E). Finally, it is well-known that T-bet is the key transcription factor for differentiation of the Th1 cell line; thus, YY1 knockdown was performed in an ex vivo Th1 subset differentiation system to determine the effect of YY1 on Th1 differentiation. Unexpectedly, the proportions of CD4^+^IL-17A^–^IFN-γ^+^ cells were not changed in the LV-YY1-shRNA–transfected group (Figure 4, F and G), which suggested that YY1 was specific for pTh17 differentiation. Overall, these data demonstrated that YY1 could specifically affect the Th17 cell pathogenic program partly through T-bet.

### YY1 regulates T-bet by binding to the promoter region and interacting with T-bet protein.

**Figure 5:**
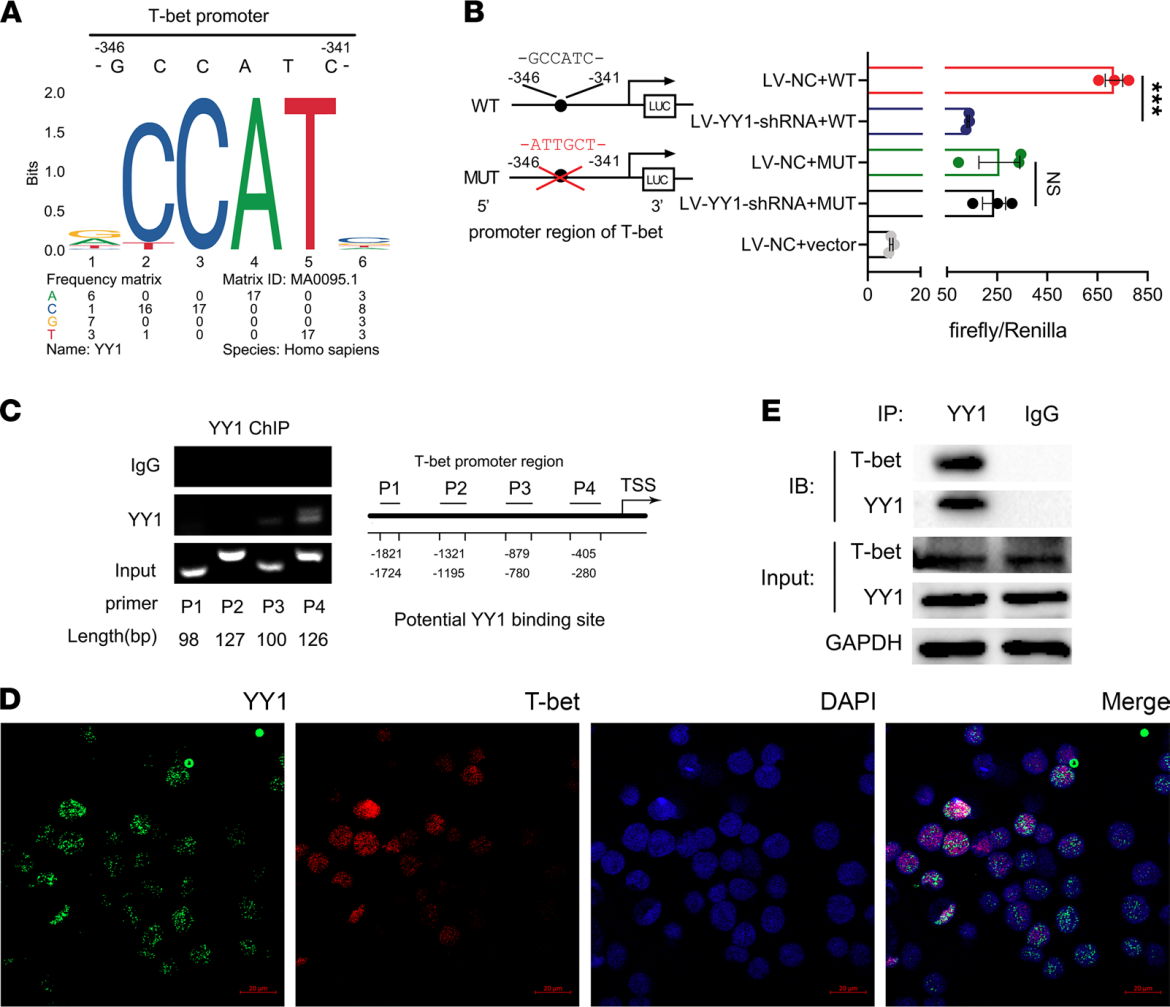
YY1 regulates T-bet by binding to the promoter region and interacting with T-bet protein. (**A**) Sequence logo representing the consensus YY1 binding motif obtained from JASPAR database. (**B**) Luciferase activity of HEK293T cells cotransfected with different promoter constructs of T-bet and lentivirus containing YY1 shRNA (LV-YY1-shRNA) or empty vectors (LV-NC). Data presented as mean ± SEM (*n* = 3 independent experiments). ***P < 0.001 (Student’s *t* test). (**C**) ChIP assay for verifying direct interaction between T-bet gene and YY1 antibody. The illustration in the right panel represents the chromatin loci associated with YY1 for ChIP (data from 2 independent experiments). (**D**) Representative confocal images of the distribution of YY1 protein (immunofluorescence staining for green) and T-bet protein (immunofluorescence staining for red) in pTh17-polarized group in vitro (scale bars = 20 μm, data from 2 independent experiments). (**E**) Co-IP analysis to confirm in vivo interactions between YY1 and T-bet protein (data from 2 independent experiments).

Next, we further elucidated the exact molecular mechanism of how YY1 regulated the differentiation of pTh17 cells through T-bet. We investigated first whether the transcriptional ability of YY1 was related to the transcriptional initiation of T-bet. The PROMO online tool and the JASPAR database were used to predict the potential transcription factor binding sites of YY1 on the promoter region of the T-bet gene. As shown in Figure 5A, CCAT was identified as the transcription factor binding site motif of YY1 and the most conserved binding site located at –346 to –341 site of the T-bet promoter region. To verify the reliability of the prediction results, T-bet promoter cDNA harboring wild-type (WT) or mutant (MUT) YY1 binding site was subcloned into pGL3 vectors. The results of the dual luciferase reporter assay revealed that YY1 knockdown decreased the luciferase activity of T-bet–WT in HEK293T cells, and the effect was resisted by mutation of the predicted site (Figure 5B). We also performed ChIP assay to further verify YY1 interaction with the promoter region of T-bet, and DNA gel electrophoresis indicated that the fragment containing the predicted T-bet *cis*-acting element position (–405 to ~–280) showed significant enrichment as compared to other control fragments (–1821 to ~–1724, –1321 to ~–1195, and –879 to ~–780) (Figure 5C).

The cellular localization of T-bet and YY1 proteins in the pTh17 polarization environment was also verified. Double immunofluorescence staining was performed and the pictures acquired with a fluorescence confocal microscope demonstrated that T-bet colocalized with YY1 in nuclei of pTh17 cells, which implied that YY1 may directly bind with T-bet (Figure 5D). Subsequently, a coimmunoprecipiation (Co-IP) assay was conducted. As shown in Figure 5E, Western blot analysis confirmed the interaction of T-bet and YY1 proteins. Collectively, these data suggested that YY1 could bind to the promoter region of the T-bet gene and interact with the T-bet protein to have an effect on pTh17 cell differentiation.

### miR-124-3p targets the 3′-UTR of YY1 mRNA.

**Figure 6:**
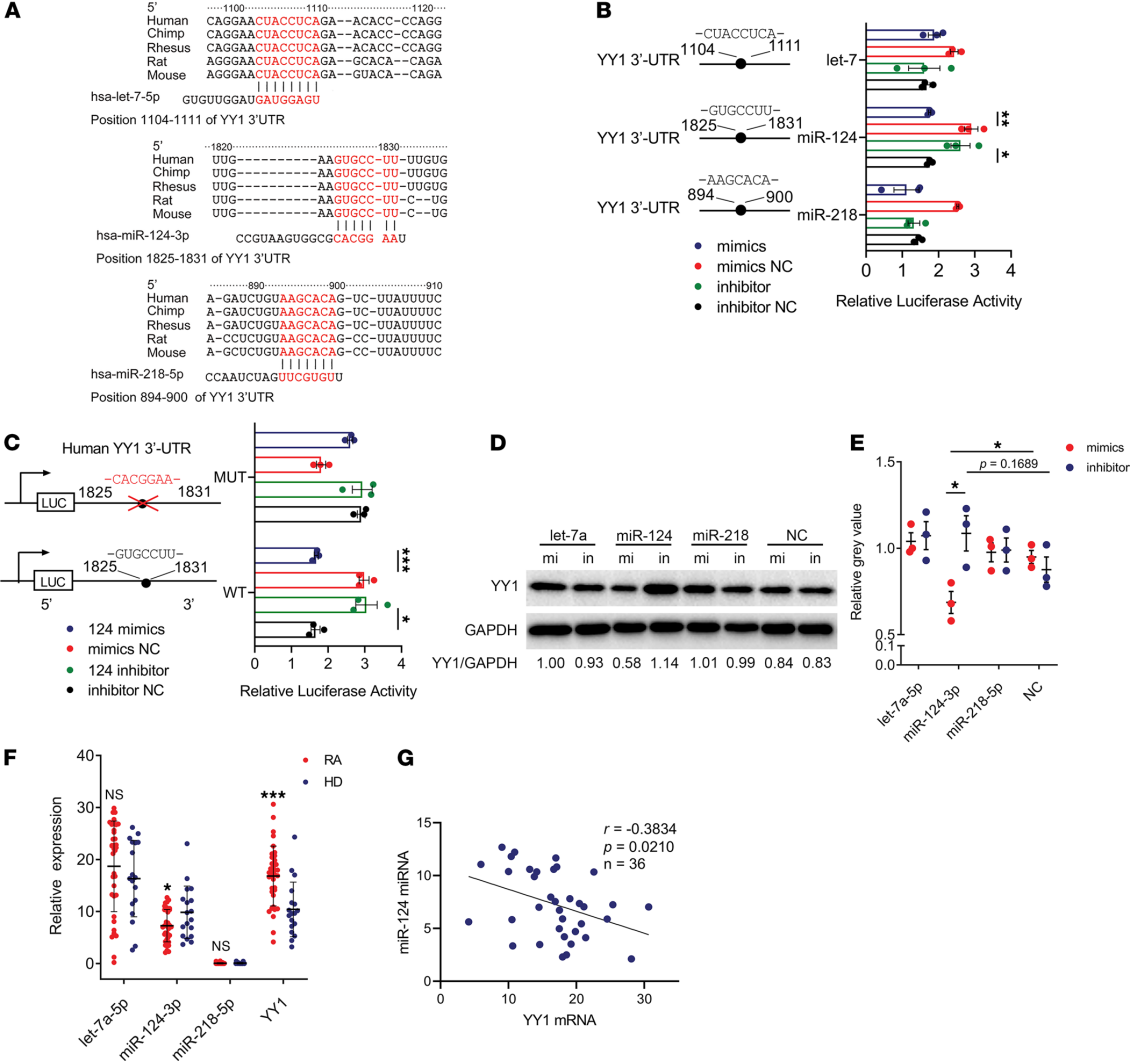
miR-124-3p targets the 3′-UTR of YY1 mRNA. (**A**) Diagrams of predicted miRNA target sites in the 3′-UTR of YY1. The solid lines represent miRNA-mRNA interactions as predicted by TargetScan. (**B**) Luciferase activity of HEK293T cells cotransfected with the pmirGLO vectors fused to the 3′-UTR of YY1 mRNA and predicted miRNAs mimics, inhibitors, or respective control plasmid. Data presented as mean ± SEM (*n* = 3 independent experiments). **P < 0.01, *P < 0.05 (Student’s *t* test). (**C**) Luciferase activity of HEK293T cells cotransfected with miR-124-3p mimics, inhibitors or respective control plasmid together with the vectors containing mutant or wild-type binding sites of miR-124-3p in YY1 3′-UTR. Data presented as mean ± SEM (*n* = 3 independent experiments). *P < 0.05, ***P < 0.001 (Student’s *t* test). (**D**) Representative Western blots of YY1 in HEK293T cells transfected with miRNAs mimics, inhibitors, or respective control plasmid and (**E**) the grayscale analysis of Western blot analysis results. Data presented as mean ± SEM (*n* = 3 independent experiments). *P < 0.05 (Student’s *t* test). (**F**) The relative gene expression levels of miRNAs and YY1 in purified CD4^+^ T cells of patients with RA (*n* = 36) and HD (*n* = 18). Data presented as mean ± SD. ***P < 0.001, *P < 0.05 (Student’s *t* test). (**G**) Analysis of the correlation of YY1 mRNA and miR-124-3p in purified CD4^+^ T cells of RA patients (*n* = 36, Pearson correlation).

From the above experiments, we had confirmed that YY1 could promote pTh17 cell differentiation in RA. However, which biomolecule can regulate YY1 in RA remains unknown. As miRNAs have been identified to widely regulate gene expression at the posttranscriptional level, we posited that there must be some specific miRNAs that could control YY1 expression. Thus, we used the miRNA target prediction program TargetScan to search candidate miRNAs for YY1. Among the predicted targets, let-7-5p, miR-124-3p, and miR-218-5p were selected based on bioinformatics predictions and the conservation pattern observed in mammals (Figure 6A). To clarify which miRNA could bind to the 3′-UTR of YY1, the pmirGLO vectors inserted with the fragment of the 3′-UTR of YY1 mRNA (YY1-let-7-5p-WT, YY1-miR-124-3p-WT, or YY1-miR-218-5p-WT) and let-7a-5p, miR-124-3p, and miR-218-5p mimics or inhibitors were chemically synthesized. We first cotransfected the miRNA mimics and the pmirGLO vectors into HEK293T cells to screen the potential miRNAs targeting YY1, followed by validation using the miRNA inhibitor/pmirGLO vector cotransfection experiments. The results suggested that miR-124-3p mimics significantly decreased the luciferase activity of WT-miR-124-3p and this phenomenon was reversed by miR-124-3p inhibitor. However, other miRNA mimics and inhibitors had no effect on that of YY1-let-7a-5p-WT or YY1-miR-218-5p-WT (Figure 6B). In addition, the dual luciferase reporter assay system further demonstrated miR-124-3p inhibited the activity of firefly luciferase reporter containing the 3′-UTR of YY1 instead of that containing the 3′-UTR with the miR-124-3p target site mutated (Figure 6C). Moreover, Western blot analysis also showed that the protein expression of YY1 was suppressed when miR-124-3p mimics were transfected into HEK293T cells after 48 hours while the opposite result was observed when miR-124-3p inhibitor was transfected (Figure 6, D and E). Purified CD4^+^ cell samples from patients with RA and HDs were analyzed to determine the expression levels of those miRNAs and YY1. No differences in let-7a-5p and miR-218-5p levels were observed. As expected, we found that the expression of miR-124-3p was downregulated in purified CD4^+^ cells of patients with RA while the expression of YY1 was upregulated compared with HDs (Figure 6F). Moreover, Pearson correlation analysis suggested that there was a negative correlation between the gene expression level of YY1 and miR-124-3p in purified CD4^+^ cells of RA (Figure 6G).

### miR-124-3p was involved in the pathogenicity of Th17 cells.

**Figure 7:**
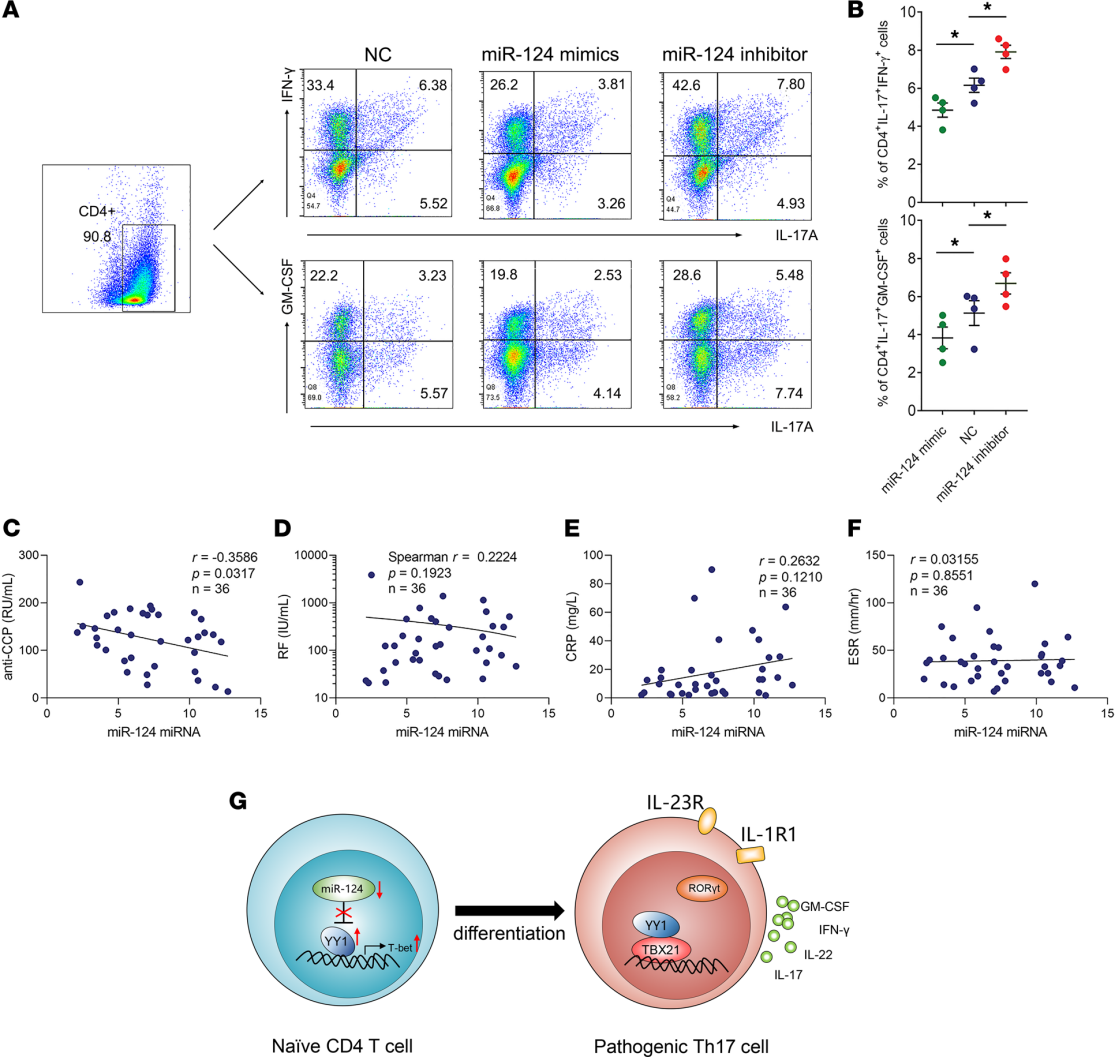
miR-124-3p is involved in the pathogenicity of Th17 cells. (**A** and **B**) Representative images of flow cytometry results for the proportion of CD4^+^IL-17A^+^IFN-γ^+^ cells and CD4^+^IL-17A^+^GM-CSF^+^ cells in pTh17-polarized group transfected with miR-124-3p mimics, inhibitors, or control plasmid. Data presented as mean ± SEM (*n* = 3 independent experiments). *P < 0.05 (ANOVA). (**C**–**F**) Analysis of the correlation of miR-124-3p and anti-CCP, RF, CRP, and ESR in purified CD4^+^ T cells of patients with RA (*n* = 36. **C**, **E**, and **F** using Pearson correlation, **D** using Spearman correlation). (**G**) Sketch of miR-124-3p and YY1 in differentiation of pTh17 cells in RA.

miRNAs have also been reported to be involved in the pathogenicity of Th17 cells (21, 22); thus, miR-124-3p mimics or inhibitors were transfected into the pTh17-polarized group. Flow cytometric analysis showed there was a reduction in the proportions of CD4^+^IL-17A^+^IFN-γ^+^ cells and CD4^+^IL-17A^+^GM-CSF^+^ cells in the pTh17-polarized group transfected with miR-124-3p mimics compared with the group transfected with miR-124-3p inhibitors or the negative control (Figure 7, A and B). Furthermore, given the potential value of miRNAs in clinical applications, a correlation analysis between miR-124-3p and RA-related clinical laboratory parameters was also performed. As shown in Figure 7, C–F, there was a significant negative correlation between miR-124-3p and anti–cyclic citrullinated peptide antibody (anti-CCP) while no obvious correlations were found between miR-124-3p and rheumatoid factor (RF), C-reactive protein (CRP), or erythrocyte sedimentation rate (ESR). In short, these data demonstrated that miR-124-3p negatively regulated the expression of YY1 by directly targeting its 3′-UTR in RA and might play a role in the pathogenicity of Th17 cells.

## Discussion

Pathogenic Th17 cells are considered to be involved in RA pathogenesis. Targeting pTh17 cells or associated molecules may be a potential strategy for clinical diagnosis, treatment, and prognosis of RA (24, 25). In our previous study, we observed that transcription factor YY1 was overexpressed in RA patients and promoted Th17 cell polarization by regulating IL-6 (16). However, what role YY1 plays in the pathogenicity of Th17 cells and whether other molecules are involved in the regulation of YY1 function remain unclear.

In this study, we first assessed the proportion of pTh17 cells in peripheral blood of patients with RA. As we expected, YY1 expression level and the proportion of pTh17 were increased in patients with RA compared with HD and OA individuals, which demonstrated pTh17 cells indeed engaged in RA development. In order to clarify what possible molecular mechanisms were implicated in the pathogenicity of Th17 and whether YY1 was associated with this process, we established an ex vivo Th17 subset differentiation system. In in vitro experiments, we observed that in comparison with the non-pTh17-polarized group or nonpolarized group, the YY1 protein level was elevated in the pTh17-polarized group, which implied YY1 was more likely to participate in pTh17 differentiation. Subsequently, YY1 shRNA lentivirus was used to knock down YY1 in the pTh17-polarized group, and we found the proportion of pTh17 cells induced in vitro was reduced. Moreover, to confirm the role of YY1 in the Th17 cell pathogenic program in vivo, we treated CIA mice with the sh-YY1 lentivirus. Indeed, blocking YY1 expression attenuated inflammation and decreased the proportion of pTh17 cells in CIA mice. In brief, our above results demonstrated that YY1 participated in RA pathogenesis.

How does YY1 influence pTh17 differentiation in RA? With the aim to elucidate this question, we comprehensively analyzed the gene expression of cells in the pTh17-polarized group using mRNA array analysis followed by qPCR validation experiments and finally identified a potential YY1 target, T-bet, which was the key transcription factor for pTh17 cell polarization. In addition, rescue experiments also confirmed that overexpression of T-bet rescued the proportions of pTh17 cells induced in vitro. Accumulating evidence indicates that pTh17 cells can express a Th1-like phenotype (26), which confers the Th17 lineage with more pathogenic potential. STAT4- and T-bet–deficient mice are protected from autoimmunity associated with Th17 pathogenesis, suggesting Th1-related transcription factors play key roles in the pathogenicity of Th17 cells (27). In addition, given T-bet is the key transcription factor for the Th1 cell line, we also knocked down YY1 in the ex vivo Th1 subset differentiation system. Nevertheless, no significant results were found between the Th1-polarized group and the respective control group. This result suggested the relationship between YY1 and pTh17 differentiation was specific.

In order to clarify more detailed mechanisms, our next step was to examine how YY1 functions on T-bet. Considering that YY1 is a bidirectional transcription factor involved in the transcriptional regulation of approximately 10% of the total mammalian genome (13), ChIP and dual luciferase reporter assays were performed to verify whether YY1 possessed transcriptional activation capability on the T-bet gene. The results demonstrated that YY1 could bind to the promoter region (–346 to –341) of the T-bet gene. Moreover, double immunofluorescence staining and Co-IP assay showed that YY1 and T-bet proteins were colocalized in nuclei of pTh17 cells, which suggested that YY1 may interact with T-bet protein to regulate downstream pathways. Together, we revealed that YY1 specifically influenced the Th17 cell phenotype and pathogenicity partly through binding to the promoter region of T-bet gene and interacting with T-bet protein.

The complex regulatory networks composed of miRNAs and related genes are implicated in various aspects of normal biological functions and life activity (18). In this study, we identified that miR-124-3p could target and regulate YY1 transcription by complementarily binding to the 3′-UTR of YY1 mRNA. In RA, we observed that the expression of miR-124-3p was downregulated in purified CD4^+^ cells isolated from PBMCs of patients and negatively correlated with the expression of YY1 and the clinical biomarker anti-CCP, implying that miR-124-3p might be a potential biomarker for clinical diagnosis of RA.

It has been well documented that miR-124-3p takes part in the regulation of the immune system and immune-related diseases (28). miR-124-3p has been reported to promote differentiation of naive CD4^+^ T cells into Th1 and Th17 cells by suppressing SOCS5 expression (29) and repressing Th17 cells through inhibiting IL-6 signaling in CIA mice treated with 1,25-dihydroxyvitamin D_3_ (30). Moreover, hybrid micelles containing methotrexate-conjugated polymer and miR-124 achieved an enhanced synergistic efficacy and showed good therapeutic effects in an adjuvant-induced arthritis rat model, although the specific and detailed therapy mechanism was still unclear (31). In fact, to our knowledge, this is the first report that YY1 regulated by miR-124-3p plays a role in the differentiation of pTh17 cells in RA.

Over the past decades, clinical trials have been demonstrated that IL-17A blockade as a monotherapy lacks good clinical efficacy in RA (32, 33), implying that not all IL-17A–producing Th17 cells induce inflammation and promote disease development. There is a possible reason that not all Th17 cells participate in RA pathogenesis; only the part of Th17 cells characterized by expressing high levels of GM-CSF and IFN-γ may be pathogenic. In addition, given the emerging data that non-pTh17 cells do not possess proinflammatory functions and in fact inhibit autoimmune inflammation (34), a promising therapeutic strategy for the future may be selected on the basis of the molecular mechanisms that regulate Th17 cell plasticity and pathogenicity, including proinflammatory cytokines, key transcriptional regulators, upstream and downstream signal molecules, noncoding RNA, and so forth. Blockade of GM-CSF would fit ideally in this approach, but the evidence to support the therapeutic effect in patients with RA is still scarce; the safety and efficacy need to be further confirmed (35). For example, IL-23 is indispensable for maintaining pathogenicity of Th17 cells while IL-23R is highly expressed in pTh17 cells (36). However, treatment with monoclonal anti–IL-12/23 p40 antibody (ustekinumab) or monoclonal anti–IL-23 antibody (guselkumab) is not very effective in RA (37). Therefore, although targeting critical factors regulating Th17 cell plasticity and pathogenicity has already become a new research trend in the field of RA treatment, more research is still needed. The findings in our study may have significant implications for the understanding of what molecular mechanism promotes differentiation of pTh17 cells in RA. By understanding these regulatory mechanisms, new therapeutic strategies targeting the balance between pTh17 and non-pTh17 cells may be a promising approach to improve the treatment of RA patients with poor response to current treatments.

In summary, our study demonstrated that YY1 negatively regulated by miR-124-3p specifically promoted pTh17 cell differentiation in RA through binding to the promoter region of the T-bet gene and interacting with T-bet protein (Figure 7G). Targeting YY1 or miR-124-3p might be a potential therapeutic strategy for the treatment of RA.

## Methods

### Patients and HDs.

A total of 82 patients with RA according to the criteria of the 2010 American College of Rheumatology/European League Against Rheumatism (38) were included in this study. In addition, 36 patients with OA who met the American College of Rheumatology guidelines (39) and 64 HDs were included as controls. Clinical characteristics of the selected participants are indicated in Supplemental Table 1. PBMCs from patients with RA and OA as well as from HDs were freshly isolated by Ficoll density gradient (Solarbio), and CD4^+^ T cells were purified from PBMCs of patients with RA and HDs using magnetic beads following the manufacturer’s instructions (Miltenyi Biotec).

### Mice.

Animal experiments were performed in accordance with the animal care and use committee guidelines. Six- to eight-week-old male DBA/1J mice (*n* = 4–5 per group) were purchased from the Shanghai Laboratory Animal Center, Chinese Academy of Science. Mice were bred and maintained under sterile and specific pathogen–free conditions and housed in cages under controlled conditions of 12-hour light/12-hour dark and with food and water ad libitum at 25°C. All animals were housed and experiments were conducted in the Experimental Animal Center of Fujian Medical University.

### Establishment and treatment of CIA.

The murine CIA model was induced as described previously (16). Briefly, the male DBA/1J mice (age, 6–8 weeks) were allocated randomly according to the average starting weight of each group and injected intradermally with 150 μg chicken CII (Chondrex) in 0.05 M acetic acid emulsified in complete Freund’s adjuvant for the first immunization. On day 21, the secondary immunization was performed with 75 μg CII in incomplete Freund’s adjuvant. Severity of inflammation was evaluated using a blind method by an independent observer who did not know the grouping of the mice. The scoring range for each mouse was 0–16, as described (24). On day 35, mice were administrated with YY1 shRNA lentivirus (LV-YY1-shRNA) or control lentivirus (LV-NC) via caudal vein, 1 × 10^8^ transducing units/mouse. The score of inflammation and joint damage were investigated until day 61, and the mice were sacrificed by cervical dislocation for further analysis. Animals were anesthetized with 20% urethane (1 g/kg) prior to the experimental procedure.

### Cell proliferation assay.

Mouse mononuclear cells were collected from spleens of the lentivirus-treated CIA mice. Cells (10^5^ per well) were incubated in the presence of CII (20 mg/mL) for antigen challenge. Cultures were maintained at 37°C in 5% CO_2_. for 72 hours. Then 5 mg/mL MTT was added and incubated for 4 hours to detect cell proliferation. The absorbance was read at 590 nm.

### H&E staining.

The CIA mice were sacrificed and the joints were removed. Next, specimens were fixed in 10% phosphate-buffered formalin and decalcified for 30 days in 10% EDTA. After decalcification, the joints were embedded in paraffin, and histological examination was performed using H&E staining.

### In vitro purification and polarization of naive CD4^+^ T cells.

Human naive CD4^+^ T cells from PBMCs of healthy honors were purified following manufacturer’s instructions (Miltenyi Biotec). The purity of isolated cells was routinely assessed by flow cytometry, and only a CD4^+^CD45RA^+^ cell suspension (catalog 17-0049-42/CD4, catalog 11-0458-42/CD45RA, eBioscience) with a purity at least 90% was used for experiments (Supplemental Figure 2). Purified naive CD4^+^ T cells at 2 × 10^5^ cells per well were cultured in 96-well, round-bottom plates with the following antibodies and cytokines for each Th subset-polarizing condition in RPMI-1640 with 10% fetal bovine serum (FBS) (both from Gibco): Th0, 2 μg/mL anti-CD3/CD28 (Gibco), 100 U/mL IL-2 (Peprotech); non-Th17, 2 μg/mL anti-CD3/CD28 (Gibco), 100 U/mL IL-2 (Peprotech), 10 ng/mL IL-6 (Peprotech), 0.5 ng/mL TGF-β (Peprotech), 5 μg/mL anti–IL-4 (Bio X Cell catalog BE0240), 5 μg/mL anti–IFN-γ (Bio X Cell catalog BE0245); pTh17, 2 μg/mL anti-CD3/CD28 (Gibco), 100 U/mL IL-2 (Peprotech), 10 ng/mL IL-6 (Peprotech), 10 ng/mL IL-1β (Peprotech), 10 ng/mL IL-23 (Peprotech), 5 μg/mL anti–IL-4 (Bio X Cell), 5 μg/mL anti–IFN-γ (Bio X Cell); Th1: 2 μg/mL anti-CD3/CD28 (Gibco), 100 U/mL IL-2 (Peprotech), 10 ng/mL IL-12 (Peprotech), 10 μg/mL anti–IL-4 (Bio X Cell).

The polarized pTh17 subsets or Th1 cells were cultured with YY1 shRNA lentivirus (LV-YY1-shRNA, MOI = 20), with control lentivirus (LV-NC, MOI = 20) or without lentivirus (control) after cytokine stimulation for 24 hours. Subsequently, cells were grown for 3 days at 37°C in 5% CO_2_. On day 4, cells were collected for qPCR and Western blot assay or stimulated with Leukocyte Activation Cocktail for 5 hours in the presence of GolgiPlug according to the manufacturer’s protocol (BD Biosciences) for the flow cytometric analysis.

### Flow cytometry.

In vitro polarized human naive CD4^+^ T cells or PBMCs freshly isolated from human participants or CIA mice after stimulation for 5 hours with Leukocyte Activation Cocktail as described above were first prepared. Stimulated cells were first incubated with PBS containing 1% bovine serum albumin (BSA) and 2% FBS for 45 minutes to block Fc receptors and then stained for the exclusion of dead cells using LIVE/DEAD Fixable Dead Cell Stain Kit (Invitrogen). Next, cells were surface stained for CD4 (catalog 17-0049-42/anti-human, catalog 25-0042-82/anti-mouse), fixed, permeabilized following staining for intracellular cytokines IL-17A (catalog 11-7179-42/anti-human, catalog 45-7177-82/anti-mouse), GM-CSF (catalog 12-7209-42/anti-human, catalog 12-7331-82/anti-mouse), and IFN-γ (catalog 45-7319-42/anti-human, catalog 11-7311-82/anti-mouse) with the Fixation/Permeabilization Solution Kit (BD Biosciences) according to the manufacturer’s instructions. Unstained, isotype, and single positive controls were used for gating and compensation. All the antibodies mentioned above were purchased from eBioscience. Finally, flow cytometry was conducted on a Navios cytometer and analyzed using FlowJo software.

### RNA isolation and real-time qPCR.

Total RNA was isolated using TRIzol Reagent (Ambion) based on phenol/chloroform method. According to the manufacturer’s instructions, cDNA of mRNA-encoded genes was synthesized using the RevertAid First Strand cDNA Synthesis Kit (Thermo Fisher Scientific). cDNA of miRNAs was synthesized using the miRNA-specific stem-loop reverse transcription primer and miRNA 1st Strand cDNA Synthesis Kit (Vazyme). SYBR Green qPCR was executed using TB Green Premix Ex Taq II (Takara Biotechnology). Primers were designed using Oligo7 software or obtained from GenBank, and they are listed in Supplemental Table 2. Quantitative PCR was performed using a QuantStudio DX and the results were analyzed using QuantStudio Real-Time PCR software (Applied Biosystems).

### Western blot analysis.

Denatured protein samples were subjected to 10% SDS-PAGE (Beyotime). Proteins were then transferred onto 0.2 μm PVDF membranes (Merck KGaA) and blocked with 5% skim milk for at least 1 hour at room temperature. Next, the membranes were incubated overnight at 4°C with the primary antibody as indicated: 1:1000 YY1 antibody (Cell Signaling Technology catalog 46395), 1:1000 T-bet antibody (Cell Signaling Technology catalog 13232), and 1:2000 GAPDH (Cell Signaling Technology catalog 5174); 1:2000 β-actin (Cell Signaling Technology catalog 3700). Diluted HRP-conjugated secondary antibodies (anti-rabbit/anti-mouse IgG, 1:2000, Beyotime catalog A0208/catalog A0216) were added to appropriate immunoblots and incubated at room temperature for at least 1 hour. Finally, immunoblots were visualized by ECL reagents (Beyotime). Immunoblot images were quantified and analyzed using Image Lab software (Bio-Rad Laboratories).

### ELISA.

Serum was collected from the lentivirus-treated CIA mice, and IFN-γ or IL-17A levels were determined by ELISA using the Mouse IFN-γ (or IL-17A) Quantikine ELISA Kit according to the manufacturer’s instructions (R&D Systems).

### Microarray analysis.

Total RNA of pTh17 cells induced in vitro, described above, treated with lentivirus of LV-YY1-shRNA or LV-NC was extracted using the standard TRIzol method for microarray (Gene Expression Omnibus GSE184979). Affymetrix array hybridization and scanning were performed using GeneChip primeview human by Genechme Company Limited. All microarray experimental results have been deposited into the Gene Expression Omnibus database under accession number GSE184979.

### Lentivirus preparation.

To knock down YY1 expression, human or mouse YY1 lentiviral shRNAs were constructed from GenePharma using pGLVH1/GFP vector. The coding sequence regions of the TBX21 gene (accession number NM_013351.2) were obtained from the National Center for Biotechnology Information database, and T-bet–overexpressing lentivirus was also constructed from GenePharma using LV5 vector. The sequences of human YY1 lentiviral short hairpin RNAs were obtained directly from our previous study (16) and other sequences are designed in this study. All sequence information used was shown in Supplemental Table 3.

### Luciferase reporter assays.

The pmirGLO vectors were constructed by GenePharma Company and contain WT let-7-5p, miR-124-3p, miR-218-5p, or mutant miR-124-3p putative binding sites in YY1. The synthesis of oligonucleotides and plasmids was also conducted by the GenePharma Company. HEK293T cells (ATCC) were cotransfected with human let-7-5p, miR-124-3p, and miR-218-5p mimics or inhibitor and the pmirGLO vector containing corresponding miRNA binding sites, respectively. Cotransfection was performed using Lipofectamine 2000 (Invitrogen) according to the manufacturer’s protocol. The details of the sequences inserted into the pmirGLO vector and the sequences of miRNAs mimics as well as inhibitor are provided in Supplemental Table 3. Firefly luciferase activity was measured after 48 hours posttransfection and was normalized to Renilla luciferase activity using the Dual Luciferase Assay Reporter kit (Vazyme).

T-bet promoter regions were amplified from human genomic DNA, and the PCR products were subcloned into promoter vector pGL3-Basic (Promega) using XhoI/KpnI restriction sites to generate T-bet-WT and T-bet-MUT plasmids. The T-bet-WT and T-bet-MUT sequence information are listed in Supplemental Table 3. HEK293T cells were infected with lentivirus of LV-YY1-shRNA or LV-NC (MOI = 20) and 5 mg/mL of polybrene. Twenty-four hours after lentiviral shRNA transfection, T-bet-WT or T-bet-MUT plasmids were cotransfected into HEK293T cells using Lipofectamine 2000 with the pRL-TK vector. Cell lysate was collected 48 hours after the second transfection, and luciferase activities were measured using the Dual Luciferase Assay Reporter kit.

### ChIP assay.

ChIP assay was performed using a commercially available kit (Beyotime) according to the manufacturer’s instructions. Briefly, HEK293T cells (1 × 10^6^/immunoprecipitation) were cross-linked in 1% formaldehyde for 10 minutes at 37°C, and the reaction was quenched with 0.125 M glycine for 5 minutes at room temperature. DNA was sheared to 200–1000 bp using sonications. For IP, rabbit anti-YY1 antibody (Cell Signaling Technology catalog 46395) was used to capture the chromatin and incubated overnight with the chromatin fragments at 4°C while rabbit IgG (Beyotime catalog A7016) was used as control. On day 2, immunoprecipitated chromatin was collected and de-cross-linked for 4 hours at 65°C, then purified and quantified by PCR analysis. The primers used are shown in Supplemental Table 4.

### Double immunofluorescence.

Double immunofluorescence staining was performed for colocalization studies. Briefly, pTh17 cells induced in vitro were fixed with 4% paraformaldehyde for 20 minutes and washed with PBS several times. A mixture of 2 primary antibodies, YY1 (mouse derived, 1:400, Santa Cruz Biotechnology catalog D2419) and T-bet (rabbit derived, 1:400, Cell Signaling Technology catalog 13232), in 5% BSA-PBS-Tween (BSA-PBST) was then incubated overnight at 4°C. After washing with PBS on day 2, the cells were incubated with a mixture of 2 secondary antibodies in 5% BSA-PBST for 1 hour at room temperature in the dark (Alexa Fluor 488 goat anti-mouse IgG [H +L] and Alexa Fluor 594 goat anti-rabbit IgG [H +L], both diluted 1:200, Invitrogen catalog A-11001 and catalog A-11012). After washing, nuclei were counterstained with DAPI for 20 minutes. The results were examined using a fluorescence confocal microscope. Image acquisition and processing were performed using Zen software (Carl Zeiss Microscopy GmbH).

### Co-IP.

Endogenous protein-protein interaction in cells was examined by Co-IP experiments performed using a Pierce Classic Magnetic IP/Co-IP Kit following the manufacturer’s protocol (Thermo Fisher Scientific). Briefly, HEK293T cells were lysed with cell lysis buffer containing 1 mM PMSF. Lysates were centrifuged (12,000*g*, 4°C, 10 minutes) and collected. Then, 10% supernatant was subjected to input assays, and the remaining part was used for the next IP. Cell lysate samples and the capture antibody (YY1, 1:400, Cell Signaling Technology catalog 46395) were then incubated together overnight at 4°C with continuous mixing. On day 2, 25 μL of Pierce Protein A/G Magnetic Beads were added into the immunoprecipitated complexes and incubated for 1 hour at room temperature. The magnetic beads were collected, washed in washing buffer several times, and then eluted with 100 μL of elution buffer. Magnetic separation was performed again and supernatant was collected. Immunocomplexes in the supernatant were analyzed by Western blot as described above.

### Laboratory analysis.

Clinical data were obtained from medical records and the laboratory system. Serum anti-CCP was measured using a commercial ELISA kit (EUROIMMUN); serum levels of CRP and rheumatoid factor were measured by immunoturbidimetric assay (Dade Behring, now Siemens). ESR was determined using Westergren’s method.

### Statistics.

Mapping and all statistical analyses were conducted using GraphPad Prism 8.0 (GraphPad Software, Inc.) or R language (Version R 4.0.3). The data were presented as mean ± SD or SEM. For intergroup comparison, a 2-sample Student’s *t* test or Wilcoxon’s rank-sum test was used depending on whether data conformed to a normal distribution while correlation analysis was performed by Pearson analysis or Spearman analysis. For comparison among multiple groups, 1-way ANOVA was performed followed by Tukey’s multiple-comparison test. Heatmap analysis was performed using pheatmap package in R. All statistical tests were 2 tailed, and *P* < 0.05 was considered significant (*P < 0.05, **P < 0.01, ***P < 0.001, ****P < 0.0001).

### Study approval.

All study protocols were approved by the Institutional Medical Ethics Review Board of the First Affiliated Hospital of Fujian Medical University, Fuzhou, China (MTCA, ECFAH of FMU [2015]084-1). All participants provided written informed consent and were identified by number. All animal experiments were performed according to the committee guidelines and approved by the Animal Experiment Center of the Fujian Medical University (SYXK [Fujian] 2016-0006).

## Author contributions

All authors were involved in drafting the article or revising it critically for important intellectual content, and all authors approved the final version to be published. QO had full access to all the data in the study and takes responsibility for the integrity of the data and the accuracy of the data analysis. Jinpiao Lin and JT share the first authorship due to equal essential contributions and are ordered alphabetically by last name. They developed the concept, conceived the experiments, conducted the majority of the experiments, and completed the manuscript. YH and Junyu Lin participated in the experiments and helped with manuscript preparation. ZY and RJ helped with animal housing and collected samples. BY revised the manuscript.

## Supplementary Material

Supplemental data

## Version 1 11/22/2021

Electronic publication
