# Supplementary material for: YY1 regulation by miR-124-3p promotes Th17 cell pathogenicity through interaction with T-bet in rheumatoid arthritis
Source: JCI Insight. 2021 Nov 22;6(22):e149985. doi: 10.1172/jci.insight.149985 (PMC8663781; doi:10.1172/jci.insight.149985)
Supplement: Supplemental data [file jciinsight-6-149985-s075.pdf]

Supplementary Table 1

Participants' information and clinical characteristics.

Characteristics	RA	OA	HD
Number	82	36	64
Age (years)[a]	55(45.25,62.25)	52.5(35, 62.25)	50(45.75, 52.75)
Sex (M/F)	20/62	10/26	18/46
Anti-CCP(IU/ml)[a]	130.6(79.65,174)	/	/
RF((IU/ml)[b]	123.5(48.63,383.3)	/	/
CRP(mm/h)[b]	18.18±21.03	/	/
ESR(mg/ml)[b]	39.28±24.3	/	/

[a] Expressed as the median (25th to 75th percentile).

[b] Expressed as the means ± SD (standard deviation).

Abbreviations: RA, rheumatoid arthritis; OA, osteoarthritis; HD, healthy donors; anti-CCP, anti-cyclic citrullinated peptide antibody; RF, rheumatoid factor; CRP, C-reactive protein; ESR, erythrocyte sedimentation rate.

Supplementary Table 2

Primers used for qPCR.

Species	Gene Name	Sequence (5′-3′)
Homo sapiens	YY1	Fw: AAAACGACACCAACTGGTTCATAC<br>Rv: AAGTCCAGTGAAAAGCGTTTCC
	T-bet	Fw: AACCCAGTTCATTGCCGTGAC<br>Rv: ATGGACTCAAAGTTCTCCCGGAA
	IL-17A	Fw: GCTGGAGAAGATACTGGTGTC<br>Rv: TAATGAGTTTAGTCCGAAATGAGG
	IL-22	Fw: AAATAACAATTAGATGCCCCA<br>Rv: TAAACAAAAGTGGCATTGGT
	Foxp3	Fw: CAAGTTCCACAACATGCGACCC<br>Rv: TCCAGCTCATCCACGGTCCAC
	RORγt	Fw: GGCACCCTACCCTTTACCTG<br>Rv: TCTTGGCCTTCATTGTACCCT
	STAT3	Fw: TCTGCCTGTTTCTGTAAGCAA<br>Rv: AAAGGCTATGCTGATACAGT
	Runx1	Fw: TTCTGAAAAGCACCATTAGCC<br>Rv: AAACAAATGTATACGCTACGG
	GAPDH	Fw: CACATGGCCTCCAAGGAGTAA<br>Rv: TGAGGGTCTCTCTCTTCCTCTTGT
	miR-124-3p	Fw: CGTAAGGCACGCGGTGAA<br>Rv: AGTGCAGGGTCCGAGGTATT<br>Rt:GTCGTATCCAGTGCAGGGTCCGAGGTATTCGCACTGGATACGACTTGGCA
	miR-218-5p	Fw: GCGCGTTGTGCTTGATCTAA<br>Rv: AGTGCAGGGTCCGAGGTATT<br>Rt:GTCGTATCCAGTGCAGGGTCCGAGGTATTCGCACTGGATACGACACATGG
	let-7-5p	Fw: GCGCGTGAGGTAGTAGGTTGT<br>Rv: AGTGCAGGGTCCGAGGTATT<br>Rt:GTCGTATCCAGTGCAGGGTCCGAGGTATTCGCACTGGATACGACAACCAC
	U6	Fw: AACGCTTCACGAATTTGCGT<br>Rv: CTCGCTTCGGCAGCACA<br>Rt: CTCGCTTCGGCAGCACA

Abbreviations: Fw, Forward primer; Rv: Reverse primer; Rt: Reverse transcription primer.

Supplementary Table 3

Primers used for plasmids cloning.

Species	Primer Designation	Sequence (5′-3′)
Homo sapiens	LV-YY1-shRNA	Fw: GATCC-**GACGACGACTACATTGAACAA-** TTCAAGAGA-**TTGTTCAATGTAGTCGTCGTC**- TTTTTTG
		Rv: AATTCAAAAAAG-**CCTCCTGATTATTCAGAATAT-** TCTCTTGAA-**ATATTCTGAATAATCAGG**-AGGCG
	WT-T-bet promoter region	Fw: CGGGGTACCGGAGAAAGAGGGCAACCCG
		Rv: CCGCTCGAGCTGTCACTAGAGTCGCAGCG
	MUT-T-bet promoter region	Fw: CACTTGG**ATTGCT**TCGGAAGGCTTCCTGTAGGAG
		Rv: TCCGA**AGCAAT**CCAAGTGGGACTCGCTGGGCAT
	YY1-let7-WT	AACAGGCATCCCGAGTTCAGGAACTACCTCAGAACA CCCCAGGCCAGGTTGGTCATAGGCT
	YY1-miR-124-WT	CCCTGTGGCTCTGTGGAATTTTGAAGTGCCTTTTGTGA ATCATGAATGAAACATTTAACTT
	YY1-miR-218-WT	GAATATGGCAGAACAAGATCTGTAAGCACAGTCTTAT TTTCTTTTGTTGTCCAGAATACT
	YY1-miR-124-MUT	CCCTGTGGCTCTGTGGAATTTTGAACACGGAATTGTG AATCATGAATGAAACATTTAACTT
	let-7-5p mimics	UGAGGUAGGAGGUUGUAUAGUU
	miR-124-3p mimics	UAAGGCACGCGGUGAAUGCCAA
	miR-218-5p mimics	UUGUGCUUGAUCUAACCAUGU
	let-7-5p inhibitor	AACUAUACAACCUCCUACCUCA
	miR-124-3p inhibitor	UUGGCAUUCACCGCGUGCCUUA
	miR-218-5p inhibitor	ACAUGGUUAGAUCAAGCACAA
	NC mimics	UUCUCCGAACGUGUCACGUTT
	NC inhibitor	UUGUACUACACAAAAGUACUG
Mus musculu s	LV-YY1-shRNA907	Fw: GATCC-**GGATACCTGGCATTGACCTCT**- TTCAAGAGA-**AGAGGTCAATGCCAGGTATCC-** TTTTTTG
		Rv: AATTCAAAAAAG-**GATACCTGGCATTGACCTCT-** TCTCTTGAA-**AGAGGTCAATGCCAGGTATC**-CG
	LV-YY1-shRNA1009	Fw: GATCC-**GCCCTCATAAAGGCTGCACAA-** TTCAAGAGA-**TTGTGCAGCCTTTATGAGGGC**- TTTTTTG
		Rv: AATTCAAAAAAG-**CCCTCATAAAGGCTGCACAA-** TCTCTTGAA-**TTGTGCAGCCTTTATGAGGG**-CG

Abbreviations: Fw, forward primer; Rv: reverse primer; WT, wild-type; MUT, mutant; LV, lentivirus; shRNA, short hairpin RNA; NC, normal control.

Supplementary Table 4

Primers used for ChIP PCR.

Primer	Sequence (5′-3′)	Product Size(bp)	Position on T-bet Gene Locus
P1	Fw: GGCAGAAACTTCCCTGTTCCT	98	-1821~-1724
	Rv: CTGGTACTGTCATGTATCCGGT		
P2	Fw: AAATTATATATTTGTGCATGTGT	127	-1321~-1195
	Rv: GTGGAATTTGGGGTGATTTA		
P3	Fw: CACCCTCTCATGTAAGGCTTG	100	-879~-780
	Rv: GGTTGACTTTCAGGCAAGGAA		
P4	Fw: CTTCTAGTGTGCCCGTGCTC	126	-405~-280
	Rv: GAGACTTCAAAGCTGGGCTGA		

Abbreviations: Fw, forward primer; Rv: reverse primer.

Supplementary Figure 1

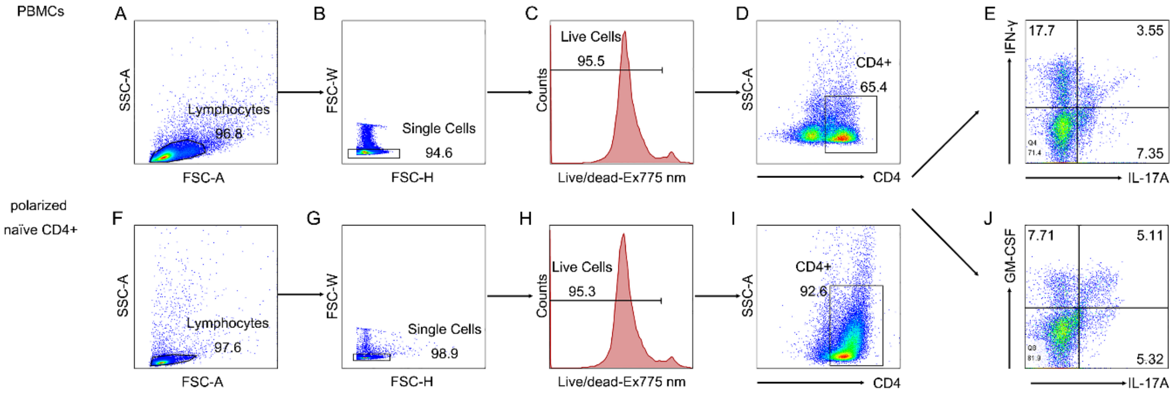

Supplementary Figure 1. Representative scheme showing the flow-cytometry gating strategy used for the analysis of $CD4^{+}IL17A^{+}IFN\text{-}\gamma^{+}$ cells or $CD4^{+}IL17A^{+}GM\text{-}CSF^{+}$ cells. The proportions of $CD4^{+}IL17A^{+}IFN\text{-}\gamma^{+}$ cells or $CD4^{+}IL17A^{+}GM\text{-}CSF^{+}$ cells were determined from PBMCs or polarized naïve $CD4^{+}$ cells. Cells were gated by FSC-A/SSC-A (A and F) to exclude debris and then by FSC-H/FSC-W (B and D) to exclude cell doublets. Next, Dead cells were excluded by live/dead staining (C and H) and $CD4^{+}$ cells ($CD4^{+}$/SSC-A) were further gated (D and I). Lastly, cells to be analysed were gated by IL-17A and IFN-γ/GM-CSF (E and J) to determine the cell proportions.

Supplementary Figure 2

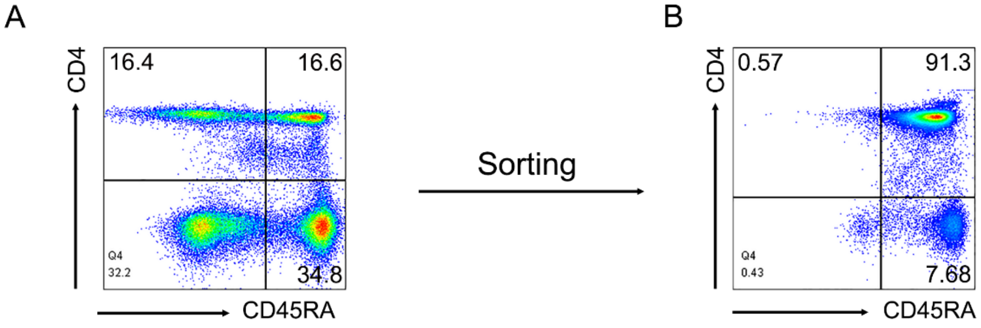

Supplementary Figure 2. The sorting efficiency of $CD4^{+}CD45RA^{+}$ cells from PBMCs. Human naïve $CD4^{+}$ T cells from PBMCs (A) were purified following manufacturer's instruction. Purity check was performed after sorting and sorted fractions with ≥ 90% purity (B) were used for further experiments.
